# Cluster randomised trial of a school-community child health promotion and obesity prevention intervention: findings from the evaluation of *fun ‘n healthy in Moreland!*

**DOI:** 10.1186/s12889-017-4625-9

**Published:** 2017-08-03

**Authors:** Elizabeth Waters, Lisa Gibbs, Maryanne Tadic, Obioha C. Ukoumunne, Anthea Magarey, Anthony D. Okely, Andrea de Silva, Christine Armit, Julie Green, Thea O’Connor, Britt Johnson, Boyd Swinburn, Lauren Carpenter, Graham Moore, Hannah Littlecott, Lisa Gold

**Affiliations:** 1 Jack Brockhoff Child Health and Wellbeing Program, Centre for Health Equity, Melbourne School of Population and Global Health, University of Melbourne, VIC, 3010 Australia; 2 Family and Community Support Services, Merri Health, Level 2, 110 Chifley Drive, Preston, VIC 3072 Australia; 3 NIHR CLAHRC South West Peninsula (PenCLAHRC), University of Exeter Medical School, University of Exeter , Heavitree Road, Exeter, EX1 2LU UK; 4 Nutrition and Dietetics, Flinders University, GPO Box 2100, Adelaide, South Australia 5001 Australia; 5 Early Start Research Institute, University of Wollongong, Northfields Ave, University of Wollongong NSW, Wollongong, 2522 Australia; 6 Institute for Safety, Compensation and Recovery Research (ISCRR), 222 Exhibition Street, Melbourne, 3000 Australia; 7 Murdoch Childrens Research Institute, Parenting Research Centre and Department of Paediatrics, University of Melbourne, VIC, 3010 Australia; 8 Body Image and Health Promotion Consultant & Educator, c/- Post Office, Repton, NSW, 2454 Australia; 9 Global Obesity Centre, Deakin University, 221 Burwood Hwy, Burwood, VIC 3125 Australia; 10 School of Population Health, University of Auckland, Private Bag 92019, Auckland, 1142 New Zealand; 11 DECIPHER, School of Social Sciences, Cardiff University, 1-3 Museum Place, Cardiff, CF10 3XQ Wales; 12 School of Health and Social Development, Deakin University, Geelong, Australia; 13 School of Public Health and Preventive Medicine, Monash University, Commercial Road, Melbourne, VIC 3004 Australia

**Keywords:** Child obesity prevention, Schools, Cluster RCT

## Abstract

**Background:**

Multi-level, longer-term obesity prevention interventions that focus on inequalities are scarce. *Fun ‘n healthy in Moreland!* aimed to improve child adiposity, school policies and environments, parent engagement, health behaviours and child wellbeing.

**Methods:**

All children from primary schools in an inner urban, culturally diverse and economically disadvantaged area in Victoria, Australia were eligible for participation. The intervention, *fun ‘n healthy in Moreland!,* used a Health Promoting Schools Framework and provided schools with evidence, school research data and part time support from a Community Development Worker to develop health promoting strategies. Comparison schools continued as normal. Participants were not blinded to intervention status. The primary outcome was change in adiposity. Repeated cross-sectional design with nested longitudinal subsample.

**Results:**

Students from twenty-four primary schools (clusters) were randomised (aged 5–12 years at baseline). 1426 students from 12 intervention schools and 1539 students from 10 comparison schools consented to follow up measurements. Despite increased prevalence of healthy weight across all schools, after 3.5 years of intervention there was no statistically significant difference between trial arms in BMI z score post-intervention (Mean (sd): Intervention 0.68(1.16); Comparison: 0.72(1.12); Adjusted mean difference (AMD): -0.05, CI: -0.19 to 0.08, *p* = 0.44). Children from intervention schools consumed more daily fruit serves (AMD: 0.19, CI:0.00 to 0.37, *p* = 0.10), were more likely to have water (AOR: 1.71, CI:1.05 to 2.78, *p* = 0.03) and vegetables (AOR: 1.23, CI: 0.99 to 1.55, *p* = 0.07), and less likely to have fruit juice/cordial (AOR: 0.58, CI:0.36 to 0.93, *p* = 0.02) in school lunch compared to children in comparison schools. More intervention schools (8/11) had healthy eating and physical activity policies compared with comparison schools (2/9). Principals and schools highly valued the approach as a catalyst for broader positive school changes. The cost of the intervention per child was $65 per year.

**Conclusion:**

The *fun n healthy in Moreland!* intervention did not result in statistically significant differences in BMI z score across trial arms but did result in greater policy implementation, increased parent engagement and resources, improved child self-rated health, increased fruit, vegetable and water consumption, and reduction in sweet drinks. A longer-term follow up evaluation may be needed to demonstrate whether these changes are sustainable and impact on childhood overweight and obesity.

**Clinical trial registration:**

ACTRN12607000385448 (Date submitted 31/05/2007; Date registered 23/07/2007; Date last updated 15/12/2009).

## Background

Childhood obesity is associated with a wide range of adverse psychosocial and physical health outcomes. Development of effective intervention approaches to prevent childhood obesity continues to be a public health priority [1]. The international evidence base of the benefits of prevention is strengthening [2]. Interventions to promote health among young people have commonly been delivered via schools [3], in large part due to their potential to reach large numbers of children simultaneously. Much of the school-based obesity prevention interventions have centred around the provision of education, aimed at enhancing factors such as young people’s knowledge, attitudes and self efficacy [2]. However, given the complex aetiology of childhood obesity, interventions solely targeting these individual and intrapersonal factors are likely to have limited effects. Schools represent key micro-environments in which children spend a substantial part of their time. They also have a key role in influencing their immediate and wider communities, and are a source of support for parents and families [3]. Hence, schools provide opportunities to go beyond providing young people with education, to provide and influence contexts which are supportive of positive health and wellbeing, consistent with Ottawa Charter principles [4]. Whilst schools are limited in the extent to which they can influence the wider impact of industry and commerce, they do provide a setting within which multi-level interventionist approaches can be developed and tested to reduce unhealthy weight gain [5]. A Cochrane review of multi-level school interventions based on the WHO Health Promoting Schools Framework (HPSF) provided evidence that interventions which combine curriculum change with environmental change and engagement with parents and community can have small but significant effects on outcomes such as BMI, as well as physical activity and dietary behaviours [6].[1–48]

There are significant gaps in the evidence base for child obesity prevention interventions which: target changes in the environmental context and policies; operate in geographic areas or populations where the burden is greatest; as well as interventions or programs which are implemented beyond one year [2]. The evidence of changes in BMI is limited due to unexplained heterogeneity in study findings and likelihood of small study bias [2]. Engaging parents and communities has proved more difficult to achieve than incremental school changes such as increased focus on health topics within the curriculum [7–9]. There is a need to document the many contextual variations and small effects of multi-level interventions that may contribute to fundamental shifts in schools’ practices and interactions with other stakeholders. Furthermore, studies which imbed harm prevention within the context of obesity prevention are needed to ensure that school-based obesity prevention strategies have positive outcomes and do not increase body dissatisfaction or weight related impacts on mental health [2, 10].

It has been argued that a standardised intervention is inappropriate to deliver in a school setting because schools are complex adaptive systems and their needs and interactions with interventions vary between schools [9, 11]. The overall behaviour of the system is constantly adapting as a result of the number of decisions made every moment by many individual agents [12, 13]. Keshavarz et al. [9] demonstrate the relevance of complex systems in the development and evaluation of health promoting school interventions. The outcomes of whole of school interventions, such as the Gatehouse Project [14] and the Inclusive Study [15] are likely to be caused by a combination of all parts of the intervention and the way in which they interact with the characteristics of the complex system in which it is implemented [9].

The community-based child obesity prevention study, *fun ‘n healthy in Moreland!,* emerged from a shared interest between a university research group and a local community health service with the aim of making a difference to the adverse health outcomes experienced through child disadvantage in an inner city area of Melbourne, Australia. In this particular community context, the population was characterised by a socioeconomically and culturally diverse population, and relative socioeconomic disadvantage associated with a significantly higher prevalence of overweight and obesity (31%) [16]. Thus, *fun ‘n healthy in Moreland!* developed with a focus on the need for a complex intervention and a rigorous mixed method study design, to meet the needs of local and state-wide public health decision-makers, and to ensure that the approach and methods were suitable and inclusive within such a diverse and disadvantaged population.

*fun ‘n healthy in Moreland!* was an evidence-based, multi-level, child health promotion and obesity prevention program evaluated using a cluster randomised controlled trial study design. The 5 year study received funding to support a 3.5 year intervention program with 24 primary (elementary) schools and a mixed method approach to evaluation. The project addressed the issue of child overweight and obesity by working closely with school communities to develop integrated, multi-level interventions guided by the limited evidence available in 2005. In contrast to many previous school-based obesity prevention projects, an extensive research component was incorporated to evaluate the physical, environmental, social, behavioural and financial impacts and outcomes of the interventions.

## Methods

### Study context

The City of Moreland, a local government municipality (population of 135,205 in 2006) is located 8.5 km north west of the central business district of Melbourne, in South Eastern Australia. Of the 31 Melbourne municipalities, this area ranked seventh in social disadvantage at the time of the study.

Census data from 2011 indicated that the majority of the residents in Moreland (58%) spoke English (only) at home compared with 71% of the population average across the Melbourne Statistical District. This municipality also has one of the highest levels of residents who belong to the Catholic and Islamic faiths (36% and 10%, respectively, compared to 30% and 4% across Melbourne). However, there is marked variation in demographic and economic background across the municipality, and it has shifted over time towards a higher socio-economic profile as housing demand and inner-urban location has resulted in families with higher median incomes moving into the area [17–19].

### Governance

*fun ‘n healthy in Moreland!* was funded by three departments of the state government (Sport and Recreation, Health, and Education). Intervention staff (Community Development Workers) were employed by Merri Community Health Services^1^ and research and evaluation staff were employed by Deakin University (2004–7) – relocating to University of Melbourne (2007–9). This model ensured that Merri Community Health Services had influence and leadership on the design and implementation of the intervention, in partnership with the schools, maximising the chance of high impact and sustainability. The implementation of the trial and evaluation study was governed by a project team comprising both researchers and Merri Community Health staff who consulted regularly with school staff, families and community leaders. Key research decisions were referred to the full team of 11 investigators, including representation from each discipline and content area relevant to the conduct of the study. Additional advice was provided by an internal Merri Community Health Services Staff Advisory Committee during the development of the study, and an external committee of government stakeholders which met annually.

### Theoretical frameworks

The design and implementation of the intervention was underpinned by the WHO Health Promoting Schools Framework, an evidence informed decision making process, and the International Obesity Task Force ‘10 guiding principles for obesity prevention’, which state that health promotion initiatives be empowering, participatory, holistic, inter-sectoral, equitable, sustainable and multi-strategy. The Health Promoting Schools Framework (HPSF) is based on health promotion theory and is consistent with a socio-environmental theoretical framework [20]. HPSF has been widely used and developed to assist schools to address health issues over the past decade [6, 21]. The advantage of the HPSF is that it is designed to guide multilevel interventions to account for environmental, sociocultural and individual influences on health behaviours. It allows for a community participatory approach [22] which was extended in this study to include models of cultural competence to guide the engagement of the culturally diverse community in the Moreland area [23–25]. “Cultural and linguistic competence is a set of congruent behaviours, attitudes, and policies that come together in a system, agency, or among professionals that enables effective work in cross-cultural situations” [26].

### Intervention

Schools were supported to develop *fun ‘n healthy* programs according to the fixed requirement of a whole school combined focus on increasing fruit, vegetable and water consumption, increasing physical activity and encouraging positive self-esteem in children. Within the intervention schools, the school community determined the exact content of the program strategies, based on interventions that had demonstrated evidence of implementation or success in previous studies, or innovative programs which had a strong likelihood of success. The *fun ‘n healthy in Moreland!* study offered schools the support of Community Development Workers (CDWs) for the 3.5 year intervention period from Jan 2006 – June 2009 who acted as knowledge brokers, providing information and guiding schools' customised development of intervention program strategies and their efforts to resource them. Three full time CDWs provided support to 4 schools each in the first 2 years. This then reduced to 2 full time CDWs providing targeted support to schools based on need. This support ensured that the strategies followed health promotion principles in creating a supportive and sustainable environment, customised for the school community to achieve changes in relation to the school system, policy, curriculum, environment, and child behavior and health outcomes. The CDWs were in turn supported by the Research Program Manager (LGi) to enable shared problem solving and links with evidence-informed approaches.

The aims of the intervention were to:

1. Reduce overweight and obesity and improve child health and wellbeing
2. Improve child and family dietary intake, increase child and family physical activity and reduce child sedentary behaviours
3. Improve knowledge and skills of school staff, family and children regarding sustainable strategies for healthy eating, physical activity and environmental changes
4. Develop sustainable positive changes in school, home and community environments (system integration, policies, physical, social, and community connections)
5. Examine contextual and programmatic features of the intervention that impact on results.

Specifically, the logic of the approach was underpinned by a hypothesis that changes in the school environment in terms of policies, programs, curriculum, physical environment and parent engagement would result in changed parent and child knowledge and behaviours, and with sufficient time lead to improvements in health and wellbeing and weight status of children.

### School selection and recruitment

Schools were eligible to participate in the study if they were located in the Moreland municipality and exclusively covered the primary (elementary) school-aged group, aged 4–13 years (*n* = 36 schools). All school principals of primary schools in the Moreland municipality were contacted by phone by the Research Project Manager (LGi) and invited to participate in the study.

**Fig. 1:**
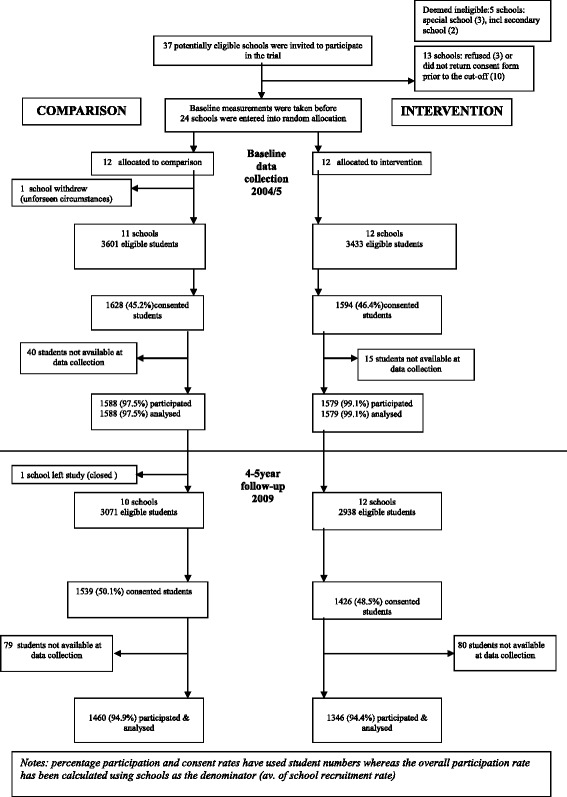
Consort flow chart

A Plain Language Statement detailing the study and research process, and a school principal consent form, were provided to all schools who expressed interest in being involved. Schools which returned the consent form were included in the study, resulting in a sample of 24 schools (65%) (Fig. 1). All children attending the consenting schools and their parent/guardian were invited to participate.

### Randomisation

Following recruitment and baseline data collection, schools were randomised using computer-generated random numbers to either actively engage with the *fun ‘n healthy in Moreland!* program (intervention arm) or continue with normal school activities and programs for healthy eating and physical activity (comparison arm). Intervention schools were provided with a memorandum of understanding which clearly articulated the parameters of the intervention and the respective rights and responsibilities of each participating organisation (school, community health service and university).

### Evaluation measures and processes

A mixed method evaluation was conducted using a repeated cross-sectional design for the collection and analysis of quantitative data. Eligible participants were in the study schools at the time of each measurement occasion at three time points: baseline (2004–5), midway (2007) and completion (2009). This contrasts with a cohort design that uses the same participants at all measurement occasions. Longitudinal analysis was only feasible on a nested sample of 350 students because of the turn-over in the school population in the 5 years of the study.

A pilot study of the data collection was initially carried out in an inner-urban, a suburban, and a rural primary school in Victoria, Australia in 2003 to test the feasibility and acceptability of the processes and measurement tools. The tools were subsequently refined for the main study collection of data at both school and individual child/parent levels Child questionnaires were completed by children in grades three to six (approximate age range 8 to 12 years). Two versions of the questionnaires were produced with one tailored to grade three and four children, the other for grade five and six children. A body image sensitivity protocol was also developed to minimize any potential harm in relation to body dissatisfaction [10].

The individual measures were as follows:

The pre-specified primary outcome, ***BMI*** was measured by:

- **BMI z-score** calculated using direct measure of child height and weight to generate BMI, and then z-scores against the WHO reference curves [27]. Project staff were trained in standardised child height and weight measurement and a process developed that was sensitive, confidential and avoided value judgements [10]. Weight in light clothing without shoes was recorded to the nearest 0.1 kg using digital scales and height to the nearest 0.5 cm using rigid stadiometers. All measures were taken twice and the mean value used. Where two readings differed by more than 0.4 kg or 4 cm, a third reading was taken and the two closest values used to calculate the mean.

***Fruit and vegetable intake and sweet drink consumption*** were measured by:

- ***Parental report*** through parent questionnaires covering issues such as family food habits [28], and usual intake of fruit, vegetable, dairy and drink consumption [29]
- ***Child report*** through child questionnaire assessing food behaviours [30]
- ***Direct Assessment of school foods:*** Lunch box survey whereby fruit, vegetables and drinks in children’s lunchboxes were recorded
- **24-h food record** [31] which was distributed to parents on a weekday for description of food and drink consumed by child for the next 24 h period.

***Participation in sedentary activity, physical activity and activity intensity*** was measured by:

- ***Parental report*** in parent questionnaires covering issues such as family physical activities and child sedentary and physical activities and level of active transport *(*Physical activity questions changed from baseline to follow up to reduce burden and increase comprehensibility)
- ***Child report*** through child questionnaire covering issues such as family physical activities and child sedentary and physical activities and level of active transport *(*Physical activity questions changed from baseline to follow up to reduce burden and increase comprehensibility)

***Child experience*** was measured by:

- ***Child-report*** through child questionnaire of quality of life using the 10-item version of the international self-reported measure of quality of life, KidScreen [32]
- ***Child focus groups*** to explore children’s concepts of health and strategies to promote health in the home and school environments

***Impacts on the school, home and community environments*** were measured by:

- ***School reported*** audit of the school food and physical activity environment, including physical activity facilities, canteen and fundraising policies and practices [33]
- ***Principal exit interviews*** to identify barriers and enablers to the school experience and likelihood of sustainability
- ***Teacher-reported*** school- and class-based nutrition and physical activity initiatives and level of support
- ***Observational measure*** **:** SOPLAY (System for Observing Play and Leisure Activity in Youth) [34] based on momentary time sampling techniques using systematic and periodic scans of individuals and contextual factors within pre-determined target areas. The instrument permits comparison of physical activity levels in different play environments [35].
- ***Process evaluation using monitoring maps, photos, and audits*** to track and record changes in school plans, policies and environment, stability of changes, costs of changes, and level of independence from the research team
- ***Parental report*** through parent questionnaire of parent and spouse/partner demographics and funds expended on nutrition and on physical activities.

An outline of these measures and data collection time points is presented in Table 1. This paper will present results from the anthropometric measures, school questionnaire, principal interviews, parent questionnaire, child questionnaire and lunchbox survey collected at baseline and completion.

**Table 1:** Outline of study measures

Measures	Description	2004	2007	2009
Child Measures				
Child Anthropometry:	Weight	✓	✗	✓
	Height	✓	✗	✓
	Child waist circumference	✗	✗	✓
Child Questionnaire	Child PA levels	✓	✗	✓
	Dietary and PA knowledge and attitudes	✓	✗	✓
	Health and wellbeing	✓	✗	✓
Lunchbox Audit	Dietary intake	✓	✗	✓
Food Record	Dietary intake	✓	✗	✗
Child Focus Group	Child perceptions of health and aspects of school programs/environment	✓	✗	✗
Parent Measures				
Parent Questionnaire	Parent knowledge and attitudes about food and PA	✓	✗	✓
	Home food and PA environment	✓	✗	✓
	Indication of the cost and time impacts of food and PA	✓	✗	✓
	Child and parent’s eating and PA behaviours	✓	✗	✓
	Socio-demographics	✓	✗	✓
School Measures				
So Play	Indication of activity levels in the playground	✓	✓	✓
Photos of Play Areas	Play equipment in the school grounds	✓	✓	✓
Teacher Questionnaire	Staff knowledge of healthy eating and PA guidelines	✓	✗	✓
School Questionnaire	Profile of school food & PA environment	✓	✓	✓
Staff Focus Group	School information including previous activities or school culture	✓	✗	✗
Resource assessment	Assessment of level of investment in interventions in terms of money spent, staff and volunteer time	✗	✓	✓
Independent capacity	Assessment of school capacity to implement sustainable changes independently	✗	✓	✓
Kids Go For Your Life Criteria	Assessment of school achievement of State Government key health promotion program areas	✗	✓	✓
Intervention Monitoring Tool	Mapping and monitoring of school intervention implementation	✗	✓	✓
Principal Interview	Perceptions of the usefulness, acceptability, efficiency of the interventions, changes in the school and external environment	✗	✗	✓

### Blinding

The randomisation allocator was blind to school status. However it was not possible for schools and participants to be blind to allocation because of the nature of the intervention. Field staff collecting data were blind to the intervention status of each school. Data collection, however, occurred on school premises and for some schools their intervention status was obvious. Schools were de-identified at data entry prior to data being sent to the analysis team.

### Power calculation

We aimed to recruit and randomise 9 schools to each trial arm (18 schools altogether) and sample 127 children from each school at each wave. Using bmi-z score as the outcome, this is large enough to detect a difference of 0.2 with 80% power at the (2-sided) 5% level of significance, assuming a standard deviation of 0.96 and an intra-cluster (intra-school) correlation coefficient (ICC) of 0.017.

### Statistical analysis

#### Pupil level outcomes

Characteristics are summarised using means and standard deviations for continuous variables and proportions for binary variables. Intervention effects are estimated based on the intention-to-treat principle with participants and schools analysed according the trial arm they were randomised to. Descriptive adiposity scores were generated using WHO cut points, with bmi z-scores as the adiposity outcome to model the intervention effect. For continuous outcomes (e.g. bmi z-score), the intervention effect was estimated using random effects linear regression models fitted by maximum likelihood estimation to allow for clustering. For dichotomous outcomes such as prevalence of overweight/obesity, marginal logistic regression models were fitted using generalized estimating equations with information sandwich (“robust”) estimates of standard error, specifying an exchangeable correlation structure. Both crude analyses and analyses adjusted for prognostic factors were run. As the study used a repeated cross-sectional design, analyses of continuous outcomes were adjusted for baseline level of the corresponding outcome by using the mean score for the school at baseline as a predictor variable in the models. Binary outcomes were adjusted for the proportion with the characteristic of interest in the study cluster at baseline. As the baseline physical activity variables differed to those used at follow up, the baseline school means for the original measures of physical and sedentary activity [36] were used as surrogate school means in the analyses of follow-up data. Models were also adjusted for child age and sex, socio-economic position ((SEP) measured by maternal education, residential SEIFA (Australian Bureau of Statistics Socio-economic Index for Areas (SEIFA) index of relative socioeconomic disadvantage), and ethnicity (only English spoken at home). Statistical analyses were conducted with STATA 10.1 (Stata Corp LP, College Station, Tex).

#### Environmental outcomes

Community Development Workers kept records of intervention strategies implemented in schools. Descriptive statistical analyses of school policies, environments and practices were undertaken using school questionnaire data. Interviews were conducted with school principals following the collection of follow up data in order to understand the impact of the intervention on schools, principals’ views on what factors mitigated or enhanced the implementation of the intervention, recommendations in relation to future strategies, and whether the model had been acceptable to the school context and systems. An inductive, thematic analysis was conducted on the interview transcripts to generate insights into principals’ perspectives on the school experience of the study, and development and response to school policies and practices.

#### Cost outcomes

A costing of the resources invested in the intervention, including the CDW salaries, school resources and parent expenses was also undertaken. Costs incurred across all schools were split equally between the intervention schools. School-level costs were split equally across the student population. Costs were discounted at 5% and presented in 2009 Australian dollars.

#### Data sharing

Data sharing is not applicable to this article as this was not part of the original consent arrangements.

## Results

Twenty-four primary schools provided written consent to participate in the study. One withdrew prior to baseline data collection due to unforseen personal circumstances for the principal. A second school closed prior to follow-up data collection. Both were comparison schools. Figure 1 shows the school and participant flow through the baseline and final follow-up cross-sectional surveys.

### Sample

At baseline 1628 (45.2%) students from 11 comparison schools and 1594 (46.4%) students from 12 intervention schools returned consent forms for data collection. At follow-up 1539 (50.1%) eligible students from 10 comparison schools and 1426 (48.5%) eligible students from 12 intervention schools returned consent forms for data collection. Recruitment/consent rate within schools ranged from 38.6% to 64.3%. Data were collected from 1460 (94.9%) recruited comparison children and 1426 (94.4%) recruited intervention children. There was wide variation in consent rates across schools. This is likely to be due to a range of factors but did show a positive correlation with SEIFA for comparison schools. It is possible that this impacted effect estimates. At baseline there were no observed differences between trial arms in the proportion of children with overseas-born mothers, but the intervention arm had higher levels of maternal education, smaller family size, and fewer possessing a health care card. At follow-up there were no observed differences between trial arms in maternal or paternal education or the proportion of children with Australian born mothers. Smaller differences in family size, healthcare card and family employment status remained (Table 2). At school level, the intervention arm had more schools from the religious sector and a smaller mean school size. School absence on data collection days was the most common reason for missing data.

**Table 2:** Characteristics for each trial arm post intervention (2009)for school cluster and individual levels

School level characteristics	Comparison (K = 10)	Intervention (K = 12)
government schools	8	6
religious sector schools	2	6
participating students	1460	1346
Size of school (mean)	367	243
Student and household level characteristics	Comparison (*N* = 1460)	Intervention (*N* = 1346)
Mother born in Australia, %	63.3	65.9
Maternal education		
≤ grade10, %	13.1	10.0
grade 11/12 and/or, tech qualification, %	47.6	47.8
University, %	39.3	42.2
Paternal education		
≤ grade10, %	14.4	12.6
grade 11/12 and/or, tech qualification, %	47.4	49.1
University, %	38.2	38.3
Employment status^a^		
Unemployed/home duties, %	13.7	9.8
Part-time, %	9.7	11.5
Full-time employment, %	76.7	78.7
Healthcare card holder, %	34.8	28.4
Food insecurity, % (ran out of food in last 12 months)	11.2%	6.5%
Number of children in family		
1 or 2 children, %	51.5	59.1
3 or 4 children, %	39.2	36.4
5+ children, %	9.3	4.5

^a^Employment status combined maternal and paternal – at least one in part time, at least one in part time or both unemployed/home duties

### BMI and others measures of adiposity

The intervention had no significant effect on prevalence of overweight and obesity. A reduction in the proportion of overweight/obesity was seen across the whole sample between baseline^2^ and follow up. In the intervention group the proportion of overweight/obesity decreased from 39.9% (95% CI: 37.4% to 42.4%, *n* = 1458) to 35.1% (95% CI: 32.6% to 39.2%, *N* = 1315). In the comparison group this decrease was from 40.9% (95% CI: 38.4% to 43.5%, *N* = 1459) to 36.7% (95% CI: 34.2% to 39.2%, *N* = 1421).

Table 3 shows the intervention effect on adiposity values at follow up. At follow up the mean BMI z score observed was 0.68 for the intervention group and 0.72 for the comparison group. After adjustment, no significant difference was observed for mean BMI z score between the two groups (mean difference: -0.05, 95% CI: 0.019 to 0.08, *p* = 0.44). No intervention effect was observed for mean weight (kg), BMI, waist circumference or proportion of overweight and/or obesity at follow up, after adjusting for age, sex, SEP and ethnicity*.*

**Table 3:** Unadjusted and adjusted mean (sd) or proportion adiposity values (WHO cut-offs) and intervention effect post intervention (2009)

	DESCRIPTIVE						MODELS						
	Comparison			Intervention			Crude model^a^	Adjusted model^b^					
	N	mean or %	sd	N	mean or %	sd	Comparative statistic^c^	Comparative statistic^c^	95% CI			p	ICC
weight (kg)	1439	31.87	10.52	1320	31.38	10.33	−0.98	−0.56	−1.40	to	0.29	0.20	0.007
bmi	1437	17.95	3.16	1320	17.82	3.03	−0.24	−0.16	−0.51	to	0.18	0.36	0.010
bmizscore^d^	1425	0.72	1.12	1318	0.68	1.16	−0.04	−0.05	−0.19	to	0.08	0.44	0.008
waist (cm)^e^	1437	63.1	9.28	1326	62.87	8.81	−0.64	−0.72	−2.17	to	0.74	0.33	0.038
overweight/obese^d^	1421	36.7%		1315	35.1%		0.90	0.96	0.77	to	1.20	0.72	0.003
obese^d^	1421	12.1%		1315	13.5%		1.10	1.10	0.80	to	1.51	0.57	0.004

^a^Adjusted for mean weight at baseline

^b^Adjusted for school mean adiposity score at baseline, age, sex, SEP(maternal education, SEIFA), ethnicity (only English spoken at home)

^c^Odds ratio for binary outcomes and mean difference for continuous outcomes

^d^WHO reference and cut-off points

^e^Baseline school mean bmi z-score used as proxy as waist not measured at baseline

^N^sample size

^ICC^intra-cluster (intra-school) correlation coefficient

### Healthy eating behaviour

Parents of children attending intervention schools reported their child consumed a greater number of serves of fruit and vegetables a day compared with parent reports of children attending comparison schools (Table 4). The intervention effect for fruit serves remained after adjustment for SEP and ethnicity.

**Table 4:** Unadjusted and adjusted mean (sd) or proportion values for child diet, physical activity and well-being, and intervention effect post intervention (2009)

	DESCRIPTIVE				MODEL						
	Comparison		Intervention		Crude model^a^	Adjusted model^b^					
	N	mean (sd) or %	N	mean (sd) or %	Comparative statistic^c^	Comparative statistic^c^	95% CI			p	ICC
HEALTHY EATING											
serves of fruit (PQ)	937	2.37 (1.36)	1009	2.48 (1.45)	0.18	0.19	0.00	to	0.37	0.05	0.009
serves of vegetable (PQ)	905	2.03 (1.31)	965	2.15 (1.33)	0.15	0.13	−0.03	to	0.30	0.10	0.008
any soft drink/day(PQ)	971	34.0	1034	28.0	1.03	0.89	0.60	to	1.32	0.55	0.013
any fruit juice/cordial/day (PQ)	971	67.6	1035	61.4	0.86	0.86	0.66	to	1.13	0.28	0.007
> =2 glasses water/day (PQ)	968	90.7	1036	94.3	1.41	1.33	0.78	to	2.30	0.30	0.007
fruit in lunchbox/canteen order	1442	72.5	1328	78.5	1.17	1.08	0.79	to	1.47	0.62	0.005
veg in lunchbox/ canteen order	1442	23.1	1328	28.0	1.27	1.23	0.99	to	1.55	0.07	0.003
juice/cordial in lunchbox/canteen order	1442	24.1	1328	15.4	0.58	0.58	0.36	to	0.93	*0.02*	0.036
water in lunchbox/ canteen order	1442	61.2	1328	73.3	1.82	1.71	1.05	to	2.78	*0.03*	0.026
PHYSICAL ACTIVITY											
active games at lunchtime(CQ)	705	69.7	628	73.1	1.26^d^	1.51	0.84	to	2.69	0.17	0.035
outside ≥ 2 h after school yesterday (PQ)	936	11.2	1012	11.7	1.22	1.33	0.75	to	2.37	0.33	0.021
outside ≥ 2 h weekend day (PQ)^e^	936	56.2	1013	54.4	0.93	0.88	0.63	to	1.24	0.47	0.018
tv ≤2 h/week day(PQ)^e^	941	79.8	1021	82.1	1.11	0.97	0.75	to	1.25	0.81	0.003
tv ≤2 h/weekend day(PQ)^e^	977	60.3	1045	61.0	1.03	1.06	0.89	to	1.27	0.53	−0.001
WELLBEING											
global kidscreen 10(CQ)	678	51.00(9.04)	583	52.20(9.20)	0.92	1.14	−0.32	to	2.60	0.12	0.010
child general health vgood/excellent^f^	707	71.20	626	80.80	1.71	1.79	1.24	to	2.61	*0.002*	0.006

^a^Adjusted for school mean score at baseline

^b^Adjusted for school mean score at baseline, age, sex, SEP(maternal education, SEIFA), ethnicity(only English spoken at home)

^c^Odds ratio for proportions and mean difference for continuous values

^d^Not adjusted for anything (no baseline value)

^e^Physical activity variables: outside weekend, sedentary activity have proxy summary measures derived from baseline physical activity questions (CLASS matrix)

^f^Baseline school mean kidscreen added as the baseline co-variate

PQ Parent Questionnaire; CQ (Child Questionnaire)

N – sample size

ICC – intra-cluster (intra-school) correlation coefficient

P - italicised *p* values denote significance (under 0.05)

Results from the assessment of lunch box contents showed 42% lower odds (OR 0.58 [95% CI 0.36–0.93], *p* = 0.02) of including fruit juice or cordial for lunch in intervention schools compared with comparison schools. Children attending intervention schools were also more likely to include plain water in their lunch box (OR 1.82 [95% CI 1.05–2.78], *p* = 0.03) (Table 4).

### Physical activity

There was no intervention effect on self-reported levels of physical and sedentary activity.

### Overall health and wellbeing

Evidence of an intervention effect was found for the self-reported general health status item but not for mean index scores for child wellbeing (Table 4).

### Longitudinal cohort

Longitudinal analysis of the results for the nested cohort showed strongly similar results, i.e. no intervention effect on adiposity, and intervention effect on healthy eating at school.

### Environmental comparisons

Community Development Worker records showed that many of the schools chose similar intervention strategies (see Table 5). Additional details on school capacity and implementation of intervention strategies will be reported separately.

**Table 5:** Intervention strategies implemented in 4 or more schools

Goal	Intervention strategy
Physical Activity	Changed playground New sports equipment Class/school exercise sessions After school sports class Active Transport Policy – Bike sheds/racks PE teacher Ride/walk to school Soccer club clinics
Healthy Eating	Healthy lunch options (developed with children, parents &/or supplier) Healthy snacks Fruit breaks Upgraded taps School water policy & water bottles School healthy eating policy School breakfast Apple slinky machines Fruit deliveries Cooking gardens Parent nutrition information and education
Health Promotion	Teacher professional development Special events Newsletter items Healthy fundraising Curriculum changes
Wellbeing	Bullying/wellbeing policies Body image training Wellbeing programs Wellbeing officer/counsellor Wellbeing focussed curriculum

School principals were originally asked to report on whether their school had written policies relating to physical activity and the canteen. As part of the intervention process, many of the schools chose to expand their canteen policy to include a broader school-wide healthy eating policy to include strategies such as healthy fundraising, drink water policies and replacement of confectionary as in-class rewards. A question about a school-wide healthy eating policy was subsequently added to the follow up questionnaire. Intervention schools were more likely to report having a written physical activity policy at follow-up (11/12) compared with comparison schools (6/10), and to show an increase since baseline (see Table 6). Intervention schools at follow up were more likely to report have a school-wide healthy eating policy (9/12) compared with comparison schools (2/10), whereas comparison schools were more likely to report having a written canteen policy (6/10) compared with intervention schools (3/12). Of those schools that did have written policies, none of nine comparison schools ‘strongly agreed’ that either the physical activity policies or the healthy eating policies were widely or consistently implemented, whereas five of eleven intervention schools with written physical activity policies and five of nine intervention schools with written school-wide healthy eating policies ‘strongly agreed’ that policies were widely or consistently implemented.

**Table 6:** Proportion of schools with written policies at baseline and follow up

	Baseline (2004/2005)		Follow Up (2009)	
	Intervention (*n* = 12)	Comparison (*n* = 10)	Intervention (*n* = 12)	Comparison (*n* = 10)
*Physical activity policy*				
Yes	8 (66.6%)	7 (70.0%)	11 (91.7%)	6 (60.0%)
No	4 (33.3%)	-	1 (8.3%)	3 (30.0%)
Missing	-	3 (30.0%)	-	1 (10.0%)
*Canteen Policy*				
Yes	2 (16.7%)	4 (40.0%)	3 (25.0%)	6 (60.0%)
No	6 (50.0%)	2 (20.0%)	8 (30.0%)	3 (30.0%)
Missing	4 (33.3%)	4 (40.0%)	1 (8.3%)	1 (10.0%)
*Healthy Eating Policy* ^a^				
Yes	-	-	9 (75.0%)	2 (20.0%)
No	-	-	2 (16.7%)	7 (70.0%)
Missing	-	-	1 (8.3%)	1 (10.0%)

^a^Data not collected at baseline

School principals reported on whether the school followed specific student, parent and staff focussed initiatives or practices regarding physical activity and healthy eating. Intervention schools reported a greater interaction between the school and parents in terms of physical activity and healthy eating initiatives compared with comparison schools, most evident in initiatives related to the school-parent interface (Table 7). Intervention parents and children also reported being more aware of school-parent initiatives, compared to those in comparison schools.

**Table 7:** School practices post intervention (2009) (assessed for completing schools)

PHYSICAL ACTIVITY PRACTICES (only schools which completed the questionnaire included)		
*Directed at students*		
School exercise classes	5/12 (42%)	3/9 (33%)
Afterschool sport activities	9/12 (75%)	6/9 (67%)
Sport clinics run by outside clubs	11/12 (92%)	7/9 (78%)
Encouragement/merit award	8/12 (67%)	4/9 (44%)
*Directed at parents*		
Excellent parental support for physical education/sport.	10/12 (83%)	2/9 (22%)
Parent exercise groups	6/12 (50%)	1/9 (11%)
*Directed at staff*		
School support for physical education/Sport excellent	12/12 (100%)	3/9 (33%)
Sufficient resources/information on what to do	11/12 (92%)	4/9 (44%)
Implementing state government program and resources (physical activity)	11/12 (92%)	6/9 (67%)
HEALTHY EATING PRACTICES *(only schools which completed the questionnaire included)*		
*Directed at students*		
Defined school time for fruit and vegetable consumption	11/12 (92%)	7/9 (78%)
Promotion of fruit & vegetables in lunchbox	8/12 (67%)	6/9 (67%)
‘nude’ food days	8/12 (67%)	4/9 (44%)
Drink bottles in class with water only	11/12 (92%)	9/9 (100%)
*Directed at parents*		
Regular information on inclusion of fruit/veg in lunchbox	10/12 (83%)	2/9 (22%)
Regular information on school strategies for healthy eating	12/12 (100%)	3/9 (33%)
Regular general information on healthy eating	11/12 (92%)	3/9 (33%)
Parent nutrition education seminars	6/12 (50%)	2/9 (22%)
*Directed at staff*		
Sufficient support from parents	4/5 (80%)	3/5 (60%)
Sufficient resources/information on what to do (healthy eating	4/5 (80%)	3/5(60%)
Implementing state government program and resources (healthy eating)	8/12 (67%)	1/9 (11%)

### Costs

The total estimated cost (discounted) of a community development worker providing external support to schools was $55,868 per school over the full period of the study or $229 per student (2009 costs). There was no associated increase in parent-reported costs to families.

### Principal exit interviews

Overall, principals across all intervention schools reported that the intervention model trialled in *fun ‘n healthy in Moreland!* was feasible and acceptable to schools and the principals themselves. For some schools, the program acted as a catalyst and driver for changes beyond the parameters of the program. The baseline data provided by the *fun ‘n healthy in Moreland!* program were used by many principals to support their efforts to achieve change and to direct them to where the changes were needed. The principals were confident that the changes introduced as part of the *fun ‘n healthy in Moreland!* program were sustainable. There was also a willingness of principals to share ideas with other principals and in many cases to share resources.

Parents were described by principals as both a significant barrier and a significant support to introducing changes into the school, depending on the school community and the proposed strategies. For more dramatic changes some principals found it easier to announce that they would be introduced at the start of the following year. For example, one school started a new year by completely removing all of the canteen items and replacing them with only three healthy lunch options. Principals reported that the parents of children in the younger years were more accepting of change and there was a gradual acceptance of the changes across the whole school community over time so that after 3.5 years of the intervention a generational shift had occurred and for the majority of the school community the changes were actually the norm and resistance was negligible.

From the principals’ perspective, the impact of the *fun ‘n healthy* strategies and evaluation processes on student body image were low, and self-esteem was not described as a concern as it was addressed through school-wide programs. There were no targeted strategies described for reducing sedentary behaviours. Children were described in many schools as having an important role in introducing changes such as new canteen menu options and healthy fundraising options to the broader school community. For example, one school worked with their local café lunch supplier to replace the traditional canteen lunch options with healthy home cooked options such as zucchini slice. The menu was selected by the students following taste tests which helped to overcome scepticism from parents and teachers about the proposed changes. One principal referred to this as an “unanticipated outcome…a lot of kids have a lot of influence over their parents”.

Cultural diversity and mixed socioeconomic status was common across the school communities albeit with different characteristics in each school. In many cases there was low parent involvement in school activities. The *fun ‘n healthy in Moreland!* model allowed for these variations because of the capacity to customize the approach for the school community. For example, one school introduced a vegetable garden for use by the school families to grow and harvest fresh, seasonal food. The increase in self-esteem for those involved was evident in a career aspirations survey carried out by the school. In another school, car-pooling was introduced to support child participation in out-of-school sport activities.

An unanticipated outcome described by some of the program principals was the fact that following the changes there was less need for discipline and there were very positive relationships between staff and students arising from the teacher involvement in the physical activities introduced by the school as part of the program.

## Discussion

This study assessed the effectiveness of a low investment, child health promotion and obesity prevention intervention, *fun ‘n healthy in Moreland!* that aimed to improve school environments, policy development and implementation, parent engagement, health behaviours, child wellbeing and adiposity. It addressed a gap in the evidence by targeting an inner urban, culturally diverse and low socioeconomic area where children were at greatest risk of overweight and obesity.

Significant reductions in overweight and obesity were observed over time at all schools, but there was no statistically significant difference in mean BMI between trial arms at follow-up in 2009. A null effect for BMI has been shown in other large studies [2], including the very large Texas Fitness Now program [38] and the HEALTHY study [39]. Investigators on the Active for Life Year 5 (AFLY5) study advocated for the importance of studying mediation of the intervention effect and subsequent relevance of theory driven interventions following the minimal impacts of their school-based intervention undertaken in the South West of England between 2011 and 2013 [40]. Given that *fun ‘n healthy in Moreland!* took the full 3.5 years to implement a comprehensive range of intervention strategies across the schools, it is also likely that although the environmental changes were achieved, the final data collection occurred too early to detect the full extent of the impact of these hypothesised mediators on child level changes. This reflects the time needed for changes to embed within a complex system [41]. Inchley and colleagues in their process evaluation of a European Network of Health Promoting Schools in Scotland [8], note that ‘there needs to be greater recognition of the time it takes to achieve such change and the support schools need to actively engage the whole school community in pursuing the HPS ideology’(p70). Longer follow-up periods may be required to capture emergent outcomes which interact with system characteristics to become greater than the sum of interventions parts [9]. Alternatively, it could mean that the HPS approach needs to be reviewed to identify opportunities to achieve greater and more rapid change. At a broader level, the potential of school-based interventions may be limited by the fact that the major drivers of the obesity epidemic are changes in food production, marketing, and distribution that lie well beyond the purview of schools [42].

The *fun n healthy in Moreland!* intervention was intended to provide a catalyst to stimulate schools to address the integrated domains of health, education, learning and wellbeing, while acknowledging the social, cultural and other drivers that operate within a school environment. It didn’t approach the issue of obesity prevention with a defined program aiming for a quick fix, but instead acknowledged the variation in school community contexts. In doing so, it enabled schools to identify solutions that were likely to be sustainable and pragmatic, aware of the complexities that operate for school communities with different funding models and the need for a shift from awareness to policy to outcomes. This approach is consistent with the Health Promoting Schools Framework. It also incorporates the criteria for success identified in a process evaluation review of Health Promoting School studies - customising the intervention to the context, and providing ongoing training and support to teachers to develop and implement programs [7]. It foreshadowed the focus on environmental changes, equity and costs advocated in the 2011 update of the Cochrane review of interventions for preventing obesity in children [2].

The cross-sectional analyses at follow up provided evidence that children at intervention schools consumed more serves of fruit per day and parents provided lunchboxes that were more likely to include a drink of water and less likely to include a sweetened drink than children at comparison schools. In this Australian context, it is customary for children to bring a packed lunch to school and where there is a canteen or tuckshop children do make purchases, however schools do not provide lunches. Given that intervention schools tended to target healthy eating first in the development and implementation of their intervention strategies, it is not surprising that the longer period of exposure to changes in the food environment translated into changes in food behaviours, although there were no intervention effects on other measures of dietary outcomes [43].

An intervention effect in terms of physical and sedentary activity was not apparent, but the measurement of physical activity in children is problematic [44]. Children have difficulty recalling discrete episodes of physical activity and cannot accurately report frequency, intensity or duration of activity. Much of the physical activity of children is incidental activity and difficult to measure. The general activity measure, time outside after school has been shown to be a useful proxy for physical activity [45], but it does not include inside activities after school that may be ignored (eg dance/aerobics/swimming in indoor centre). The cross-sectional findings for individual behaviours and health outcomes were supported by the smaller longitudinal cohort analysis.

It is acknowledged there may have been a seasonal effect on the physical activity and eating behaviour results given that data collection extended across seasons but that this should not have differentiated between intervention and comparison schools. It is possible that as two comparison schools withdrew from the study (one after baseline data collection), there may have been an artificial inflation of the intervention effect. It is also possible that some children within the intervention schools were not exposed to intervention and conversely some children in comparison schools may have benefited from health promotion strategies employed by the school. This is positive in terms of promoting healthy school environments [9] but highlights the challenges of conducting intervention trials in community settings [46]. Matching schools on religious status or other factors could have increased power but only if the correlation between schools within matched pairs was sufficiently large to compensate for the loss of degrees of freedom incurred if a corresponding matched analysis was used. A matched sample would also have meant that if one school drops out, as did happen in this study, the matched pair would have been lost from the analysis as well.

While the specific health promoting actions of schools varied with the differing strengths, needs and culture of individual schools, we identified real changes in school policies, environments and practices to improve healthy eating and increase physical activity. Overall the proportion of intervention schools with written physical activity and school-wide healthy eating policies was markedly higher than in comparison schools. Intervention schools also instituted a greater number of health promoting practices targeting students, staff and parents. These changes represent the success of this complex intervention in achieving sustainable change in school community environments and systems. Engagement of parents is recognised as a highly challenging component of Health Promoting Schools interventions and an important characteristic of complex school systems [9].

Future evaluation is necessary to seek evidence of sustainability of changes to school policies, environments and practices. A decision was made not to strongly brand the program within the schools to allow them to ‘own’ the changes and to ensure that they continued beyond the period of the study. Exit interviews with principals of intervention schools supported this approach with demonstrated changes in many schools in the environment, programs and the social norms of the school since the commencement of the *fun ‘n healthy in Moreland!* program. Principals reported varied levels of changes from low to high impact and low to high sustainability. Many changes were less policy based and more informal in their development. There was a clear lack of strategies to reduce sedentary behaviours, perhaps because at the time of the study, the predominant sedentary activities of TV watching and video games, were less under the schools’ influence or control. Some school communities had a high proportion of families committed to healthy eating and physical activity which supported changes in the schools. Otherwise the changes were dependent on the passion and commitment of the principal and a preparedness to counter opposition from the parents. Even though the HPS approach promotes a participatory approach to decision making [21], dramatic changes occurred when there was a show of leadership by school principals. Where negotiation resulted in compromises it was less successful and had less impact. However, there were many examples of children being involved in decision making which was reported by school principals and Community Development Workers as helpful in overcoming parent and teacher scepticism. This shows the potential for child participatory methods to direct school-based changes, particularly when appropriate support is provided [47]. Linking strategies to other key messages such as an environmental policy was also found to be successful in the *fun ‘n healthy in Moreland!* trial. For example, introducing ‘Nude Food Days’ when children could only bring package free food to school reduced waste and resulted in a greater reliance on fresh, unprocessed foods. These different pathways to change and differing roles of the practitioners and stakeholders, reflect the variability and challenges inherent in a complex intervention placed within a complex system [8, 41].

A strength of the *fun ‘n healthy* intervention approach was the relatively low cost of the provision of a Community Development Worker (CDW) (equating to $229 per student across the 3.5 years of the intervention, or $65 per child per year). It was also a very flexible approach allowing schools to identify where they would best be able to instigate changes resulting in fewer demands of school time and resources. The CDW also synchronized with programs that were already funded and ongoing, such as ‘Go for your Life’, a state funded award scheme.

The cluster randomized trial design enabled the assembly of intervention and comparison groups that were similar in measureable and unmeasurable attributes. Further protection from confounding was gained as the comparison group was followed contemporaneously with the intervention group, making this study design robust to outcome variations over time. Although larger than many other school-based trials, the comparatively small number of schools in this study may have limited the detection of impacts. The smaller size of the intervention schools compared to comparison schools may have inflated the intervention effect. However, the demographic profile at follow-up was not markedly different and inclusion of socio-demographic characteristics into the multivariable analysis had little impact on the effect estimates.

This intervention study was implemented over a period of time where there was a heightened media attention on childhood obesity, an increasing investment of State Government funding into non-government organisation led initiatives for healthy eating and physical activity. Mid-way through the study period, a statewide school awards program on healthy eating and physical activity policies and behaviour change strategies was introduced [48]. This is likely to have had a contaminating effect and to have therefore lessened differences between intervention and comparison schools, highlighting how contextual factors can impact on intervention effectiveness [6].

## Conclusion

Building knowledge and expertise in preventing childhood obesity continues to be a public health priority. Despite the growing evidence from school-based interventions, the majority of studies focus on individual and intrapersonal change, often with interventions of less than one year duration. This 3.5 year intervention demonstrates that it is possible to effect system level change and some improvements in health and wellbeing outcomes from investments that focus on the school environment and aim to be long-term, evidence-based and encompassing of the complexities that are real for schools, families and in particular those who are less economically privileged. However, only limited translation of those environmental changes into improved behaviours and weight status were evident at follow up. A long term commitment to addressing the needs of school communities, and a knowledge-broker/ community development approach, is likely to be most effective in achieving policy, curriculum, behavior and health outcome change.

## Abbreviations

HPSF: Health Promoting Schools Framework
SEP: Socioeconomic Position
SEIFA: Socio-economic Index for Areas
CDW: Community Development Worker
AFLY5: Active for Life Year 5
NIHR: National Institute for Health Research.

## Footnotes

1 Recently renamed Merri Health

2 The original baseline paper for this study used IOTF cut points and so the adiposity results can’t be compared to the results reported here. There are currently two alternative obesity cut-off methods – those using IOTF cut points [49] and the more recent WHO standards for older children 27. de Onis M, Onyango AW, Borghi E, Siyam A, Nishida C, Siekmann J: Development of a WHO growth reference for school-aged children and adolescents. *Bulletin of the World Health Organization* 2007, 85(9):660–667.
